# 

**Published:** 1871-07

**Authors:** 


### EXCHANGES.

THE SEMI-CENTENNIAL REPORT of the Directors and Surgeons of the New York Eye & Ear Infirmary, is received. The institution is in a flourishing condition.

HERALD OF HEALTH, published by Wood & Holbrook, at 13 and 15 Laight St., New York, at two dollars per annum. This is the oldest health journal in the country.

GOOD HEALTH—a journal of the laws of correct living, published monthly by Alexander Moore, Boston, at two dollars a year. It is a high-toned, popular health journal, that is securing a deserved popularity.

HOME AND HEALTH—a monthly magazine, devoted to health and the home circle. W. R. DePuy & Bro., Publishers, 805 Broadway, N. Y., at $1.50 per annum—is an exceedingly neat and readable journal.

PENNSYLVANIA HOSPITAL REPORTS.—These are the most valuable reports of surgical operations, that are received by us. They are far superior to Guy's Hospital Reports, both in the value of the cases reported, and the minuteness with which they are given.

THE SECOND ANNUAL REPORT of the Maryland Eye & Ear Infirmary, of Baltimore, is received. It shows the institution to be in able hands, and is doing an immense amount of good.

"FOR EVERYBODY" is the title of an illustrated family journal, published by H. H. Sage, in Buffalo, N. Y., at one dollar and fifty cents a year. It is one of the very best illustrated journals published. We will furnish it and the BISTOURY for $1.50 a year.

VIRGINIA CLINICAL RECORD, a monthly journal of Medicine, Surgery, and the Collateral Sciences, published by M. W. Hazlewood, at Richmond, Va., for one dollar per annum. It is neat, very cheap, and exceedingly interesting and valuable to the physician.

AMERICAN JOURNAL OF MICROSCOPY.—Edited by E. M. Hale, M, D., and published by G. Mead & Co., 182 South Clark Street, Chicago. This is the only journal in America devoted to Microscopical science. It is ably edited, cleverly illustrated and well, calculated to create a popular taste for the beautiful and instructive study of microscopy. Price $2.00 per annum.

THE MEDICAL TIMES—a semi-monthly journal of medical and surgical science, published by J. B. Lippincott & Co., at 715 and 717 Market St., Philadelphia, at a yearly subscription price of four dollars—is the cheapest and most valuable medical journal that comes to our table. We recommend it to our medical readers, if they desire to read communications and reports of cases from the most eminent practitioners of our land.

WHEAT-MEAL FLOUR.—Good, sound, fresh wheat, ground in a grist-mill, forms the best flour for making family bread, but this can not always be obtained in cities. Millers are quite apt to grind into "Graham meal" their poorest wheat, such as will not make white, sweet flour. For the dfficulty you mention, bread formed of two parts of sweet flour and one part of bran, is excellent. A large part of the obstinate cases of constipation can be wholly or in part removed by a free use of bread prepared in this way. The bread is really delicious when well made. Some manufacturers prepare a cleaned bran for dietetic purposes which is very nice.—*Boston Jour. Chemistry.*

### "HOW TO MAKE IT PAY."

Read the several premium lists in this number of the BISTOURY. We offer a splendid Sewing Machine for one hundred subscribers, Webster's Unabridged Dictionary for twenty-four subscribers, and will give the BISTOURY away by clubbing with other journals.

We offer very large *Cash* premiums to those who will work for our magazine. Our journal is so cheap—only fifty cents a year—that subscribers are easily secured for it. Write for specimen copies, and our Cash Premium List.

Address:

THE BISTOURY,
Elmira, N. Y.

### AN ELEGANT PREMIUM.

We are glad we hit upon the sewing machine premium. It has been the most successful one we have ever offered. Our lady canvassers are earning a sewing machine in two or three days time.

**For One Hundred Subscribers**

at fifty cents a subscriber, we will forward to any address, safely boxed, a

**"McLean & Hooper Sewing Machine,"**

which we will guarantee to be equal, if not superior, to any machine in the market.

Only think of that! For fifty dollars we will forward one hundred copies of the BISTOURY and an elegant sewing machine!

The "McLEAN & HOOPER" makes the Elastic Lock Stitch," which will not rip, even if every third stitch be cut, and takes its thread directly from the spools—no shuttle being used. It will stitch, hem, fell, tuck, quilt, cord, bind, baste, braid, embroider and gather better than any machine in the market. As before stated, we will guarantee it to be a first-class machine in every particular.

We have had agents secure us one hundred subscribers in one single day. Our Magazine is cheap, useful, and a great help and convenience in any family. Send for sample copies—we will furnish them *free* to all who desire to canvass for it. Twenty-eight pages of reading matter in every number. Only fifty cents a year. Upon the receipt of fifty dollars we will ship, to any address, nicely boxed and packed, a "McLean & Hooper" family sewing machine, with extra needles, oil-can, screw-driver, gauge, and printed circulars complete. Be careful to give the Name, Post Office, County and State of every subscriber, plainly written. Send the money by draft, or post office order, to

THAD S. UPDEGRAFF, M. D.,
EDITOR OF BISTOURY,
ELMIRA, N. Y.

### ANOTHER PREMIUM.

WEBSTER'S DICTIONARIES.

We will cause to be forwarded, direct from the Publishers, by Express, one copy of "Webster's unabridged Dictionaries," (price, $12.00) 840 pages, 3,000 illustrations, for *fifty* subscribers to the BISTOURY. Send us $25, with the addresses in full for fifty subscribers, and we will furnish the BISTOURY for one year, to every address, giving the dictionary as a premium, to the getter-up of the Club.

For *twenty-four* subscriber at fifty cents each, we will give as a premium, one copy of "Webster's National Pictorial Dictionary," (price $6,) 1040 pages and 600 illustrations.

It will be seen that we offer nothing but first-class premiums to those who work for our Magazine. We hope in this manner to double our circulation for volume VII. Send on the subscribers and secure the premiums offered.

### THE BISTOURY GIVEN AWAY.

OUR TERMS FOR CLUBBING WITH OTHER JOURNALS,

We will cause any of the publications below to be sent, together with THE BISTOURY, for one year, on the following terms:—

| | Pub. Price | with Bistoury. |
|---|---|---|
| Good Health, | $ 2.00 | 2.00 |
| Peterson's Ladies' Magazine. | 2.00 | 2.00 |
| Phrenological Journal, | 3.00 | 2.00 |
| Herald of Health, | 2.00 | 3.00 |
| Wood's Household Magazine, | 1.00 | 1.00 |
| "Little Corporal," | 1.50 | 1.50 |
| "Our Young Folks," | 2.00 | 2.00 |
| The Scientific American, | 3.00 | 3.00 |
| American Jour. of Microscopy, | 2.00 | 2.00 |
| Medical Times, Phil'a, | 4.00 | 4.00 |

Forward the price of any of the above publications to our address, and you will receive both journals regularly, for one year.

Address,

THE BISTOURY,
ELMIRA, N. Y.

